# The Challenges and Barriers to the Integration of Laboratory Services in Iran from Laboratory Experts’ Point of View

**Published:** 2018-06

**Authors:** Ali MOUSELI, Mohammadreza AMIRESMAILI, Mohsen BAROUNI, Siamak MIRAB SAMIEE, Leila VALI

**Affiliations:** 1. Social Determinants in Health Promotion Research Center, Hormozgan Health Institute, Hormozgan University of Medical Sciences, Bandar Abbas, Iran; 2. Dept. of Health Management, Policy and Economics, Kerman University of Medical Sciences, Kerman, Iran; 3. Medical Informatics Research Center, Institute for Futures Studies in Health, Kerman University of Medical Sciences, Kerman, Iran; 4. Modeling in Health Research Center, Institute for Futures Studies in Health, Kerman University of Medical Sciences, Kerman, Iran; 5. Health Reference Laboratory Research Center, Ministry of Health and Medical Education, Tehran, Iran; 6. Environmental Health Engineering Research Center, Kerman University of Medical Sciences, Kerman, Iran

**Keywords:** Laboratory services, Integration, Networking, Iran

## Abstract

**Background:**

Laboratory services fragmentation creates problems such as non-accountability for costs and quality, not being patient-centered and unsustainability of services in long run. Therefore, health systems consider laboratory services integration an inevitable way. This study aimed to investigate the challenges and barriers to the integration of laboratory services in Iran.

**Methods:**

This qualitative case study was conducted in 2016. Using purposive sampling, semi-structured interviews were conducted with 34 informed participants. Each interview lasted between 30 to 60 min. Acceptability, transferability, reliability, and verifiability were used to assess the validity, accuracy and reliability of qualitative data. Framework approach was used to analyze data.

**Results:**

Lack of economy of scale, unfair access, lack of grading, low quality, development of national strategies to create an integrated network of laboratories, criteria of the laboratories establishment, creation of necessary infrastructure, empowering the private sector and standardization of indicators were considered the most important problems of laboratory services integration in Iran; they were classified into two main themes.

**Conclusion:**

Identified issues are challenges which adversely impact the integration of laboratory services. Therefore, providing infrastructures with increased cooperation between various organizations to increase access to laboratory services in the form of an integrated network is essential.

## Introduction

Today, health systems in different countries are faced with fragmentation (1) created enormous problems in access, quality, costs, satisfaction and utilization of resources (2). Laboratory services whose results are the basis of 60%–70% of important clinical decisions suffer from fragmentation. It causes some problems such as non-accountability for costs and quality, unsustainability of services in long run, providing services not patient-centered, absence of comprehensive information management systems and absence of integrated management; their effects are clearly visible in the effectiveness of laboratory services (3, 4).

Integrated network of laboratory services was one of the most successful strategies to overcome these problems (5, 6). This network enjoys features like comprehensiveness of provided services, a systematic and defined referral system, an updated information management system and aggregated resources (7). Integration is the ability of the health system to provide people with the required services in a fair manner to achieve optimum results and proportional to their out-of-pocket payment (8, 9). Service integration is often defined as a spectrum based on the communications degree between services or organizations ranging from mutual communication to full integration (10). Integration may take place vertically, horizontally or virtually (11).

There is no general agreement on the best model of integration suitable for different countries. The highest degree of integration i.e. full integration might be suitable for one country and at the same time inappropriate for another one. Therefore, different forms of integration in health care should be adopted based on the needs, goals, and circumstances of each country. Moreover, the level of integration should be related to the differentiation degree of services and departments which, in turn, are related to the objectives and organizational environment (12). Before any attempt at integration, it is necessary to explore the problems and possible issues.

Concerning the future plans of Iran’s health system toward integration of laboratory services (13), this study aimed to identify the challenges and barriers to integration in laboratory services in Iran.

## Materials and Methods

The present qualitative study was conducted in 2016 in Kerman, Iran. Thirty-four experts were selected purposefully. Of them, five were managers and experts from the General Directorate of reference laboratory in Ministry of Health and Medical Education in Tehran, Iran; five were managers of Iranian Association of Clinical Laboratories in Tehran, Iran; four were reference laboratory managers and experts in Kerman, Iran; and twenty were directors and pathologist in charge of clinical laboratories in Kerman, Iran. The inclusion criteria were based on their managerial experiences, deep understanding of the laboratory processes and problems of laboratories integration in Iran.

Thirty-four face-to-face semi-structured interviews were conducted. An interview guide was developed after consulting with the experts and reviewing the literature (14–16). The first two interviews were in-depth. The purpose of these exploratory interviews was to provide a better understanding of the context during interviews and to prepare a suitable set of questions for other interviews. All interviews were conducted and tape-recorded in 2016.

In addition to consents were obtained from participants, assured that their personal information would remain confidential. Each interview lasted 30–60 min. Due to the saturation of information, interviews with more people stopped.

### Analysis

To analyze the data, the Framework Analysis method was used that consisting of five steps of ‘familiarization’, ‘identification of a thematic framework’, ‘indexing’, ‘charting’, and ‘mapping and interpretation’ (17). Getting familiar with the scope and diversity of contents, all interviews were transcribed and reviewed. Based on reviewing by researchers and discussing, 54 data set and key features were selected and indexed. This dataset that formed the basis of the thematic framework was filtered by researchers’ discussing and classified on 35 codes. In next step, charting was done and mentioned 35 codes classified on four main themes based on mapping between themes and sub-themes. In the final step, codes were refined and rearranged on two main themes and nine sub-themes. Acceptability, transferability, reliability, and verifiability were used to assess the validity, accuracy and reliability of qualitative data.

### Ethical approval

The participants’ information, recorded sound files, and their names were kept secret. Written informed consent was obtained from all study participants, and they all had the right to withdraw from the study at any time.

## Results

Problems related to laboratory service integration in Iran were classified into two main themes and nine sub-themes (Table 1).

**Table 1:** Main Themes and Sub-themes of Integration Problems of Laboratory Services in Iran

***Themes***	***Sub-themes***
1. The necessity of integration	1. Lack of economies of scale in laboratories
	2. Lack of leveling by clinical laboratories
	3. Unfair access to laboratory services
	4. Low quality of laboratory services
2. Integration requirements	1. Determining the establishment criteria of the laboratories
	2. Formulating of national policies in the creation of integrated network of laboratories
	3. Creating of necessary infrastructures
	4. Determining factors affecting laboratory standardization
	5. Empowering the private sector

### Theme 1: The necessity of integration

Theme 1 classified viewpoints of laboratory service providers about necessity of integration (Table 2). Accordingly, participants stated the existing laboratories are ineffective economically. In this regard, one of the participants believed “current laboratory costs are high and do not bring economies of scale” (p. 2). Similarly, another participant stated, “non-professional behaviors of governmental systems in relation to laboratory financial issues have caused some problems; if they are not solved on time; many laboratories will experience hard conditions, and even will be bankrupt” (p. 5). According to other participants’ statement, laboratory integration can help purchase and deploy advanced laboratory devices” (p. 3), but “with integration of main and referral central laboratories, we don’t need to use high-tech devices in all hospitals”(p. 17).

**Table 2:** Sub-themes and Sub-sub-themes of the Necessity of Integration of Laboratories

***Sub-themes***	***Sub-sub-themes***
1. Lack of economies of scale in laboratories	- Dollar exchange rate and volatility
	- Public inflation
	- Exchange rate tariffs mismatch
	- Mismatch between tariffs in private and public sectors
	- Delays in payment of claims by insurance companies
	- Lack of insurance payments according to price of services
	- High price of materials and equipment
	- Lack of leveling in public and private clinical laboratories
	- Absence of pathologists in charge of health laboratory
2. Lack of leveling by clinical laboratories	- Lack of facilities and equipment in clinical laboratories
	- Scattered areas
	- Number of patients referring to laboratories
	- Laboratory distance from other centers and population covered
3. Unfair access to laboratory services	- Effect of individual, geographical, structural and financial factors
	- Low accuracy in testing
	- Lack of confidence in test results due to multiplicity of influencing factors
	- Absence of pathologists in all laboratories
	- Tariff mismatch with service costs
	- Lack of high-quality equipment and consumables due to sanctions
4. Low quality of laboratory services	- Absence of adequate supervision of suppliers, consumables, supplies and laboratory equipment in terms of price, obligations, and warranty supports
	- Lack of laboratories accountability for provided services
	- Incumbency of the government in laboratories instead of supervision
	- Inadequate physical space
	- Different instructions

Lack of leveling in laboratory services is another factor that strengthens the idea of laboratory integration. Two participants believed “owing to failure to follow leveling, laboratories act separately and do not follow the same instructions” (p. 8) and “they do various tests with different instructions due to defects in monitoring systems and evaluating the quality of services”(p. 7). One of the participants stated “creating laboratory network can improve laboratory services leveling and provide a unique trend in presenting lab services so as to prevent loss of resources and increase quality of services” (p. 11).

Fair access to laboratory services is another factor which makes integration of laboratory services inevitable. One of the participants believed “people do not trust the services provided in some laboratories; that causes test replication in several laboratories and imposes a lot of costs on people and health system” (p. 27). Another participant believed “the geographical location of laboratories has the greatest impact on access to laboratory services. People are deprived of some laboratory services because laboratories are far from them” (p. 13).

Low quality of laboratory services is another factor that makes necessity of integration inevitable. One of the participants believed “laboratories with real and single-rate tariffs will only seek to improve the quality of laboratory services”(p. 9). Another participant said, “instead of controlling all matters, the government must monitor laboratories performance, and all diagnostic services must be delivered by the integrated private sector” (p. 15).

### Theme 2: Integration requirements

Another issue of integration of laboratory services in Iran is integration requirements (Table 3). One of the integration requirements is to determine criteria for laboratory construction. One of the participants stated “despite widespread and varied geographical distribution and difference in the burden of diseases, the construction, and delivery of laboratory services are the same, and laboratories do not provide services proportional to the needs of each region; it can reduce the effectiveness of laboratory services and causes no response to fair and real needs of people” (p. 5). Another participant believed “in current laboratory construction, needs of different age groups are not considered; that it causes patients frequently visit various laboratories for a special service” (p. 1).

**Table 3:** Sub-themes and Sub-sub-themes of the Integration Requirements of Laboratories

***Sub-themes***	***Sub-sub-themes***
1. Determining the criteria of laboratories establishment	- Geographic distribution
	- Age pyramid
	- Burden of diseases
	- Type of equipment and manpower existence
	- Quality of provided services
	- Construction site of laboratories
	- Formulation and implementation of dysfunctional guidelines
2. Formulating of national policies in the creation of integrated network for laboratories	- Removing the distances and population criteria
	- Lack of implementation of land use plans for the country to assess the need for medical laboratories
	- Lack of organization of the referral system in laboratories
	- Collecting the necessary data
	- The need for creation of referral laboratories
	- The need for establishment of a referral system for clinical samples
3. Creating the necessary infrastructures	- The creation of mega-labs
	- Cost pricing of laboratory services
	- Providing efficient manpower, materials, and equipment
	- Utilizing of modern technologies
	- Adopting appropriate rules and regulations
	- Providing adequate financial resources
4. Determining factors affecting laboratory standardization	- Government’s support for laboratories with modern technologies
	- Principles of management
	- Quality control and quality assurance
	- The tariff difference between public and private sector
5. Empowering the private sector	- Lack of government’s support for private sector, currency and special equipment
	- Scrap and non-standard physical space in most laboratories
	- Poor equipment in some laboratories

Another integration requirement is the formulating of national policies in the creation of integrated network for laboratories. One of the participants believed “medical laboratories both in own policymaking and major policies are affected by the policies of Health Ministry; in the most cases, these policies are contradictory and cause confusion and personalization of laboratories”(p. 34). Another participant believed “senior officials at the Ministry of Health consider clinical laboratory as industry and this approach can impose new problems for governmental laboratories in the future” (p. 12).

Creation of the necessary infrastructure is another point in the laboratory integration requirements. Based on the views of participants, “establishing the necessary infrastructures require the use of scientific and rigorous methods to address the community needs” (p. 21), and “timely access to reliable laboratory services requires an integrated network, and coherent and organized structure for the country’s laboratories” (p. 3).

Determining factors affecting standardization of laboratories is another issue in laboratory integration requirements. In this regard, participants believed “nowadays, technologies, innovations and specialized activities are limited in governmental laboratories due to the lack of integration and networking” (p. 25) and “medical laboratories in hospitals have not equipped with modern laboratory equipment” (p. 19). Another participant believed “kits, current supplies and equipment in Iran’s laboratories aren’t standard. These factors increase the costs and repeated the tests” (p. 33). Empowering the private sector is another requirement in integration of laboratories. One of the participants believed “people are not able to pay the differences between private and public tariffs; that is why people do not refer to the private laboratories, and some of them stop working due to the lack of economies of scale” (p. 20). Other participants also believed “private laboratories do not have currency and enjoy government’s support” (p. 31) and “the private sectors are forced to reduce their costs to survive and remain in a competitive market; unfortunately, it sometimes causes scrap” (p. 23).

## Discussion

Findings of this research depicted a picture of the challenges related to the integration of laboratory services in Iran. These challenges were classified into two main themes and nine sub-themes. One of the factors was lack of economies of scale. About 5% to 25% of tests carried out in laboratories were unnecessary and duplicate due to the lack of integrated systems. Unnecessary costs can be reduced and economies of scale of laboratory services can be obtained by creating an integrated network for laboratories (5). Integrating of laboratories and forming Mega-lab could provide accessible, high quality and profitable laboratory services (18). Economies of scale were occurred in any model of network configurations of laboratories. Therefore, no matter what level of integration is done, economies of scale are the effects of laboratories integration (19).

Lack of leveling is another factor that makes integration of laboratory services essential. Lack of comprehensive referral systems in Iran led to over-consumption of services and economic inefficiency due to improper use of financial and human resources (20). In all countries especially in resource-poor ones should establish a referral system in functional network for laboratories with considering in a comprehensive national laboratory strategic plan (21).

Fair access to laboratory services is the other factor in the integration of laboratory services. Equitable, comprehensive, long-term and coordinated access to obtain treatment goals of patients was not possible in the current health system and should move towards integrated services (22, 23). The lack of access to laboratory test results was conducted in other laboratories or clinical centers, and the creation of an integrated network was one of the ways to avoid extra costs and parallel activities (24).

Another factor that is important in the integration is low quality of laboratory services. The quality of clinical effectiveness (25), length of stay (26) and the number of visits (27) were improved and medication errors were decreased (28) after creating an integrated network of services. Service provision was improved quantitatively and qualitatively, and positive results were obtained in the use of human resources, equipment, and physical space after integrating a comprehensive network of laboratories in Tabriz through integrating nine units into one main unit (29).

In this study, identified factors have been emphasized for integration requirements by several researchers. One of the integration requirements is to determine the criteria for construction of laboratories. A series of parallel laboratory structures were imposed large costs on the healthcare system of countries; to overcome this situation, integrated laboratory services are necessary for terms of managerial structure, practical educations, resource efficiency, equipment maintenance, information management, geographical location and quality management (30). In Belgium clinical laboratories of most European countries were suffering from fragmentation and duplication in laboratory services delivery and subsequent excessive consumption of resources; solve this problem is to move towards the creation of a network of laboratories, centralization of services and the use of information technology to facilitate communication between laboratories (31).

Formulating of national policies in the creation of integrated laboratory network is another requirement. Successful integration was required the necessary legislation at the macro-level, presence of strong regulatory leverage in the form of laws and regulations, single-rate tariffs, cost pricing, expanding multi-sectoral vision among managers, and guaranteeing success factors mentioned for integration (32).

According to this study, the creation of necessary infrastructures is another integration requirement. The referral system is one of the important infrastructures. The referral system as a bridge between service providing levels plays an important role in reducing costs and providing access to more specialized services. However, the system still has problems mentioned in some studies. Lack of communication between different levels, self-referential, lack of knowledge about the referral system were major obstacles in the referral system (33). Trained workforce was essential for the effective implementation of health programs (34). Having inter-sectoral and intra-sectoral relations was necessary for more effective results and more efficient and more sustainable health systems (35).

Determination of factors affecting standardization of laboratories is another integration requirement. There were different standards and protocols for quality systems of clinical and research laboratories (36). One of the standardization indicators of laboratory services was the use of requirements specified in standards of laboratory quality management system achieved in the form of laboratory service network (37). Empowering the private sector was the last requirement in the integration of laboratories in Iran. A study on outsourcing of laboratory units in one hospital of Lorestan, Iran showed positive results were achieved from the reduced costs and increased revenue (38). Reviewing the results of outsourcing Firouzgar hospital pharmacy in Tehran, Iran showed outsourcing decreased costs to zero, and the revenue of the hospital was 8200 USD a month (39).

### Limitations

This study faced some limitations. Difficulty in conducting interviews at all levels was one of the limitations; to solve this problem, documents and interviews in the media and official papers were used. This study was conducted proportional to the policies governing the health system of Iran. In order to generalize the results to other countries, it is necessary to consider their conditions.

## Conclusion

Several factors affected the integration of laboratory services. Identified key issues were challenges that strongly influenced the integration of laboratory services. Scope of this program was just too broad, and the role of each factor was necessary to achieve the planned objectives. Moreover, the mentioned issues should be considered interconnected loops in planning and implementation. Paying special attention to human resources was essential in terms of quantity and quality, and proper training of personnel in relation to the referral purposes could have a significant influence on the integration of laboratory services. In addition, trying to provide infrastructures and increased cooperation between various organizations is essential to increase access to services in the form of an integrated network of laboratory services.

## Ethical considerations

Ethical issues (Including plagiarism, informed consent, misconduct, data fabrication and/or falsification, double publication and/or submission, redundancy, etc.) have been completely observed by the authors.
